# Authors' reply

**Published:** 2008-05

**Authors:** Amitava Das, Biswarup Ray, Debabrata Das, Somnath Das

**Affiliations:** Department of Ophthalmology, RG Kar Medical College, Khudiram Bose Sarani, Kolkatta 700 004, India. E-mail: doctoramitava@yahoo.com

Dear Sir,

In response to the query raised by Dr. Taksande and his colleague[1] on our published paper,[2] we would like to clarify that:

1. The patient had mental retardation.
2. The echocardiogram with Doppler showed cardiomegaly with thickened mitral and tricuspid valves.
